# Expression of BDNF and trkB in the hippocampus of a rat genetic model of vulnerability (Roman low‐avoidance) and resistance (Roman high‐avoidance) to stress‐induced depression

**DOI:** 10.1002/brb3.861

**Published:** 2017-10-23

**Authors:** M. Pina Serra, Laura Poddighe, Marianna Boi, Francesco Sanna, M. Antonietta Piludu, M. Giuseppa Corda, Osvaldo Giorgi, Marina Quartu

**Affiliations:** ^1^ Department of Biomedical Sciences University of Cagliari Monserrato (CA) Italy; ^2^ Department of Life and Environmental Sciences University of Cagliari Cagliari Italy

**Keywords:** brain‐derived neurotrophic factor, genetic model of depression, hippocampus, immunohistochemistry, Roman rat lines, trkB, western blot

## Abstract

**Introduction:**

The selective breeding of Roman High‐ (RHA) and Low‐Avoidance (RLA) rats for, respectively, rapid versus poor acquisition of the active avoidance response has generated two distinct phenotypes differing in many behavioral traits, including coping strategies to aversive conditions. Thus, RLA rats are considered as a genetic model of vulnerability to stress‐induced depression whereas RHA rats are a model of resilience to that trait. Besides the monoamine hypothesis of depression, there is evidence that alterations in neuronal plasticity in the hippocampus and other brain areas are critically involved in the pathophysiology of mood disorders.

**Materials and Methods:**

Western blot (WB) and immunohistochemistry were used to investigate the basal immunochemical occurrence of brain‐derived neurotrophic factor (BDNF) and its high‐affinity tyrosine‐kinase receptor trkB in the dorsal and ventral hippocampus of adult RHA and RLA rats.

**Results:**

WB analysis indicated that the optical density of BDNF‐ and trkB‐positive bands in the dorsal hippocampus is, respectively, 48% and 25% lower in RLA versus RHA rats. Densitometric analysis of BDNF‐ and trkB‐like immunoreactivity (LI) in brain sections showed that BDNF‐LI is 24% to 34% lower in the different sectors of the Ammon's horn of RLA versus RHA rats, whereas line‐related differences are observed in the dentate gyrus (DG) only in the ventral hippocampus. As for trkB‐LI, significant differences are observed only in the dorsal hippocampus, where density is 23% lower in the DG of RLA versus RHA rats, while no differences across lines occur in the Ammon's horn.

**Conclusion:**

These findings support the hypothesis that a reduced BDNF/trkB signaling in the hippocampus of RLA versus RHA rats may contribute to their more pronounced vulnerability to stress‐induced depression.

## 1 INTRODUCTION

The Roman low‐ (RLA) and high‐avoidance (RHA) rat lines were bidirectionally selected from the original Wistar stock for, respectively, poor versus rapid acquisition of the two‐way active avoidance response in a shuttle‐box (Broadhurst & Bignami, 1965; Driscoll & Bättig, 1982; Giorgi, Piras, & Corda, 2007) and have become one of the most validated genetic models for the study of fear/anxiety‐ and stress‐related behaviors (Fernández‐Teruel, Escorihuela, et al., 2002).

Along the selection procedure of the Roman lines many other behavioral traits have been segregated. Thus, RHA rats display a robust sensation/novelty seeking profile, behave as active copers in the face of aversive conditions, and show high impulsivity and an innate preference for natural and drug rewards (Steimer & Driscoll, 2003; Escorihuela et al., 1999; Fernández‐Teruel, Driscoll, et al., 2002; Fattore, Piras, Corda, & Giorgi, 2009; Moreno et al., 2010; Sanna et al., 2015), whereas RLA rats behave as reactive copers, display a robust activation of the hypothalamus–pituitary–adrenal (HPA) axis when exposed to stressors, and are prone to develop stress‐induced depression (Carrasco et al., 2008; Fernández‐Teruel, Escorihuela, et al., 2002; López‐Aumatell et al., 2009; Piras, Giorgi, & Corda, 2010; Piras, Piludu, Giorgi, & Corda, 2014; Steimer, Python, Schulz, & Aubry, 2007). These findings suggest that RLA rats represent a valid genetic model to investigate the neural circuitry and molecular mechanisms underlying stress‐induced depression and, more specifically, to study depression associated with anxiety symptoms. Multidisciplinary studies aimed at identifying the central neuronal circuits involved in the above‐mentioned behavioral differences across the Roman lines have shown that their divergent phenotypic traits may be accounted for, at least in part, by differences in the functional responses of their central monoaminergic systems (D'Angiò, Serrano, Driscoll, & Scatton, 1988; Giorgi, Lecca, Piras, Driscoll, & Corda, 2003; Giorgi, Piras, Lecca, & Corda, 2005; Giorgi et al., 1994; Giorgi, Piras, et al., 2003 Giorgi et al., 2007, 2015; Lecca, Piras, Driscoll, Giorgi, & Corda, 2004; Sanna et al., 2015; Tournier et al., 2013).

In recent years, a neurotrophic hypothesis has been posited to explain the pathophysiology of depression and the mechanism of action of antidepressant drugs. According to this hypothesis, the vulnerability to stress‐induced depression is due to alterations in the expression of genes encoding trophic factors in neurons that are innervated by monoaminergic projections (Nestler et al., 2002; Stahl, 2000). In addition, the neurotrophic hypothesis of depression postulates that exposure to stress and antidepressant treatments modulate the expression of specific growth factors, such as the brain‐derived neurotrophic factor (BDNF), which support neuronal survival and promote monoaminergic and glutamatergic neurotransmission in brain regions involved in the regulation of mood and emotion (Alboni et al., 2010; Burke, Advani, Adachi, Monteggia, & Hensler, 2013; Duman, 2004; Duman, Heninger, & Nestler, 1997; Duman & Voleti, 2012; Nestler & Carlezon, 2006; Nibuya, Morinobu, & Duman, 1995; Vaidya & Duman, 2001). In particular, BDNF is a member of the neurotrophin family (Barde, Edgar, & Thoenen, 1982) that promotes neuronal viability during development and in adulthood (Ibáñez, 1995). Upon its synthesis in the neuronal cell body, BDNF is anterogradely transported to the axon terminal and released in the synaptic cleft (Altar & DiStefano, 1998; Conner, Lauterborn, Yan, Gall, & Varon, 1997; Farhadi et al., 2000). The effects of BDNF are mediated by the activation of intracellular signaling pathways upon high affinity binding to the trkB receptor, a member of the trk family of tyrosine kinase receptors (Binder & Scharfman, 2004; Reichardt, 2006).

BDNF and trkB mRNA and protein immunoreactivity are widely distributed in the central nervous system of both rats (Conner et al., 1997; Drake, Milner, & Patterson, 1999; Yan, Radeke, et al., 1997; Yan, Rosenfeld, et al., 1997) and humans (Benisty, Boissiere, Faucheux, Agid, & Hirsch, 1998; Connor et al., 1997; Phillips et al., 1991; Quartu, Lai, & Del Fiacco, 1999; Quartu et al., 2010). Under baseline conditions, BDNF and its receptor trkB are densely expressed in the hippocampal formation (Conner et al., 1997; Phillips et al., 1991; Quartu et al., 1999, 2013; Webster, Herman, Kleinman, & Weickert, 2006; Yan, Radeke, et al., 1997; Yan, Rosenfeld, et al., 1997), one of the key regions implicated in depression‐linked maladaptive neuronal plasticity (Dias, Banerjee, Duman, & Vaidya, 2003; Malberg, Eisch, Nestler, & Duman, 2000; Vaidya, Siuciak, Du, & Duman, 1999). Accordingly, the hippocampal volume is reduced in patients with post‐traumatic stress disorder (Angelucci, Brene, & Mathe, 2005), and with depression associated with the BDNF Met polymorphism, which is known to be less active than its normal variant (Autry & Monteggia, 2012). The hippocampus is functionally parcelled along its longitudinal septo‐temporal axis into the dorsal and ventral subregions: the dorsal hippocampus is preferentially involved in spatial learning and memory, while the ventral hippocampus plays a central role in the control of the stress response and anxiety (Tanti & Belzung, 2013).

Despite the numerous studies reporting on the involvement of BDNF in both depression and the therapeutic effects of antidepressants, there is a relative paucity of data regarding the localization of BDNF in the hippocampus of genetic animal models of stress‐induced depression. However, previous studies have shown that, in rats, the bilateral infusion of BDNF into the dentate gyrus of the dorsal hippocampus produces antidepressant‐like effects in behavioral depression models (Shirayama, Chen, Nakagawa, Russell, & Duman, 2002); moreover, in the dorsal hippocampus, chronic electroconvulsive treatment increases the acute electroconvulsive induction and prolongs the expression of BDNF and trkB mRNA (Nibuya et al., 1995), and also up‐regulates the expression of the gene encoding BDNF in the dentate gyrus granule cell layer (Ploski, Newton, & Duman, 2006). Hence, the present study was designed to characterize the hippocampal distribution of BDNF and its receptor trkB in RHA and RLA rats under baseline conditions, with special focus on the dorsal hippocampus, using Western blot (WB) and immunohistochemistry techniques.

## 2 MATERIALS AND METHODS

### 2.1 Animals

Sixteen outbred male adult rats from each Roman line (weighing 300–380 g), aged 4 months, were obtained from the colony established in 1998 at the University of Cagliari, Italy (Giorgi et al., 2005). Rats were housed in groups of four per cage and maintained under temperature‐ and humidity‐controlled environmental conditions (23°C ± 1°C and 60% ± 10%, respectively), under a 12‐hr light–dark cycle, with lights turned on at 8:00 a.m., and with standard laboratory food and water available ad libitum. To avoid stressful stimuli resulting from manipulation, each rat was gently handled once daily for 3 days before sacrifice and the maintenance activities in the animal house were carried out by a single attendant. All procedures were carried out in compliance with the European Union (Directive 2010/63/EU) and the Italian national guidelines and protocols (D.L. 04/04/2014, n. 26) and approved by the Ethical Committee for Animal Care and Use of the University of Cagliari (No. 18/2014). Every possible effort was made to minimize animal pain and discomfort and to reduce the number of experimental subjects.

### 2.2 Sampling

**Figure 1:**
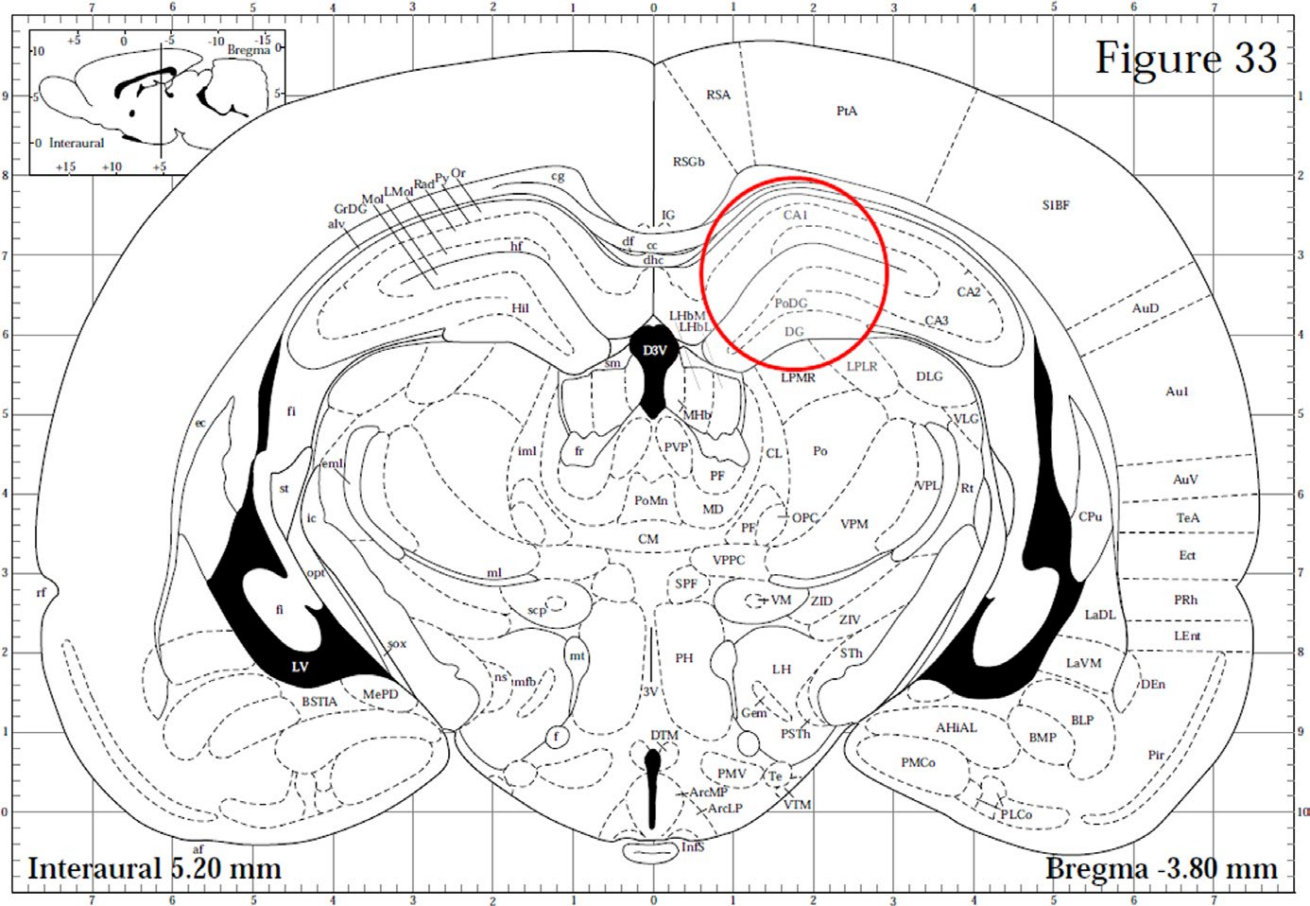
Schematic representation of a rat brain coronal section (Figure 33 from Paxinos & Watson, 1998). The red circle denotes the area of dorsal hippocampus taken for western blot analysis by means of a 2.5 mm punch

Immediately after sacrifice by guillotine, the brains were rapidly dissected and processed for either WB or immunohistochemistry. For WB, brains were cooled in dry ice for 15 s, placed in a brain matrix and cut in 2 mm thick coronal slices using the stereotaxic coordinates of the rat brain atlas of Paxinos and Watson (1998) as a reference. Bilateral punches (diameter 2.5 mm) of the dorsal hippocampus were taken as described by Palkovits (1983) (Figure 1). For each rat, tissue punches from both hemispheres were pooled, rapidly frozen at −80°C, and homogenized in distilled water containing 2% sodium dodecylsulfate (SDS) (300 μl/100 mg of tissue) and a cocktail of protease inhibitors (cOmplete^™^, Mini Protease Inhibitor Cocktail Tablets, Cat# 11697498001, Roche, Basel, Switzerland). For immunohistochemistry, brains were fixed by immersion in freshly prepared 4% phosphate‐buffered paraformaldehyde, pH 7.3, for 4–6 hr at 4°C, and then rinsed overnight in 0.1 m phosphate buffer (PB), pH 7.3, containing 20% sucrose.

### 2.3 Western blot

Total protein concentrations were determined as described by Lowry, Rosebrough, Farr, & Randall (1951) using bovine serum albumin as a standard. Proteins from each tissue homogenate (40 μg), diluted 3:1 in 4X loading buffer (NuPAGE LDS Sample Buffer 4X, Cat# NP0008, Novex by Life Technologies, Carlsbad, CA, USA), were heated to 95 °C for 7 min and separated by sodium dodecyl sulfate (SDS)‐polyacrilamide gel electrophoresis (SDS‐PAGE) using precast polyacrylamide gradient gel (NuPAGE 4%–12% Bis‐Tris Gel Midi, Cat# NP0321, Novex by Life Technologies) in the XCell4 Sure LockTM Midi‐Cell chamber (Life Technologies). Internal mw standards (Precision Plus Protein Western C Standards, Cat# 161–0376, Bio‐Rad, Hercules, CA, USA) and recombinant human BDNF protein (rhBDNF) (Cat# B‐257, Alomone Labs, Jerusalem, Israel) were run in parallel. Blots were blocked by immersion in 20 mm Tris base and 137 mm sodium chloride (TBS) containing 0.1% Tween 20 (TBS‐T) and 5% milk powder for 60 min at room temperature. The primary antibodies were rabbit polyclonal antibodies against BDNF [Cat# N‐20 sc‐546, RRID:AB_630940, Santa Cruz Biotechnology] and trkB [Cat# (794) sc‐12, RRID:AB_632557, Santa Cruz Biotechnology], both diluted 1:1000 in TBS containing 5% milk powder and 0.02% sodium azide. Incubations with primary antiserum were carried out for two nights at 4°C. After TBS/T rinse, blots were incubated for 60 min, at room temperature, with a peroxidase‐conjugated goat anti‐rabbit serum (Cat#9169, RRID:AB_258434, Sigma Aldrich, St Louis, MO, USA), diluted 1:10,000 in TBS/T. Controls for equal loading of the wells were obtained by immunostaining the membranes as above, using a mouse monoclonal antibody against glyceraldehyde‐3‐phosphate dehydrogenase (GAPDH) (MAB374, RRID:AB_2107445, EMD Millipore, Darmstadt, Germany), diluted 1:1,000, as primary antiserum, and a peroxidase‐conjugated goat anti‐mouse serum (AP124P, RRID:AB_90456, Millipore, Darmstadt, Germany), diluted 1:5,000, as secondary antiserum. In order to control for nonspecific staining, blots were stripped and incubated with the relevant secondary antiserum. After TBS/T rinse, protein bands were developed using the Western Lightning Plus ECL (Cat# 103001EA, PerkinElmer, Waltham, Massachusetts, USA), according to the protocol provided by the company, and visualized by means of ImageQuant LAS‐4000 (GE Healthcare, Little Chalfont, UK). Approximate molecular weight (mw) and relative optical density (O.D.) of labeled protein bands were evaluated by a blinded examiner. The ratio of the intensity of BDNF‐ and trkB‐positive bands to the intensity of GAPDH‐positive ones was used to compare relative expression levels of these proteins in the RHA and RLA lines. The O.D. was quantified by Image Studio Lite Software (RRID:SCR_014211, Li‐Cor, http://www.licor.com/bio/products/software/image_studio_lite/).

### 2.4 Immunohistochemistry

Coronal sections of RLA and RHA rat brains were examined in pairs on the same slide. Semiconsecutive cryostat sections (14 μm thick) were collected on chrome alum‐gelatin coated slides and processed by the avidin–biotin–peroxidase complex (ABC) immunohistochemical technique. The endogenous peroxidase activity was blocked with 0.1% phenylhydrazine (Cat# 101326606, Sigma Aldrich, St Louis, MO, USA) in phosphate buffered saline (PBS) containing 0.2% Triton X‐100 (PBS/T), followed by incubation with 20% of normal goat serum (Cat# S‐1000, Vector, Burlingame, CA, USA). The same rabbit polyclonal antibodies against BDNF and trkB (Santa Cruz Biotechnology, Santa Cruz, CA, USA) used for WB, both diluted 1:500, were used as primary antibody. A biotin‐conjugated goat anti‐rabbit serum (BA‐1000, RRID:AB_2313606, Vector, Burlingame, CA, USA), diluted 1:400, was used as secondary antiserum. The reaction product was revealed with the ABC (Cat#G011‐61, BioSpa Div. Milan, Italy), diluted 1:250, followed by incubation with a solution of 0.1 m PB, pH 7.3, containing 0.05% 3,3'‐diaminobenzidine (Sigma Aldrich, St Louis, MO, USA), 0.04% nickel ammonium sulfate and 0.01% hydrogen peroxide. All antisera and the ABC were diluted in PBS/T. Incubation with primary antibodies was carried out overnight at 4°C. Incubations with secondary antiserum and ABC lasted 60 min and 40 min, respectively, and were performed at room temperature. Negative control preparations were obtained by incubating tissue sections in parallel with either PBS/T alone or with the relevant primary antiserum preabsorbed with an excess of the corresponding peptide antigen (Cat# sc‐546P and sc‐12 P, for BDNF and trkB respectively, Santa Cruz Biotechnology, Santa Cruz, CA, USA). Slides were observed with an Olympus BX61 microscope and digital images were captured with a Leica DFC450C camera.

### 2.5 Image densitometry

For the quantitative evaluation of BDNF and trkB immunohistochemical labeling, representative 10x magnification microscopic fields, taken from sections of six animals for each line, were blindly analyzed with ImageJ (http://rsb.info.nih.gov/ij/; RRID:SCR_003070) to calculate the density of immunoreactivity per μm^2^. Mean gray values from unstained areas were subtracted from the gray values of the immunostained regions to exclude background staining.

### 2.6 Statistical analysis

WB and immunohistochemical data were statistically evaluated using the Student's *t* test for independent samples.

## 3 RESULTS

### 3.1 Western blot

**Figure 2:**
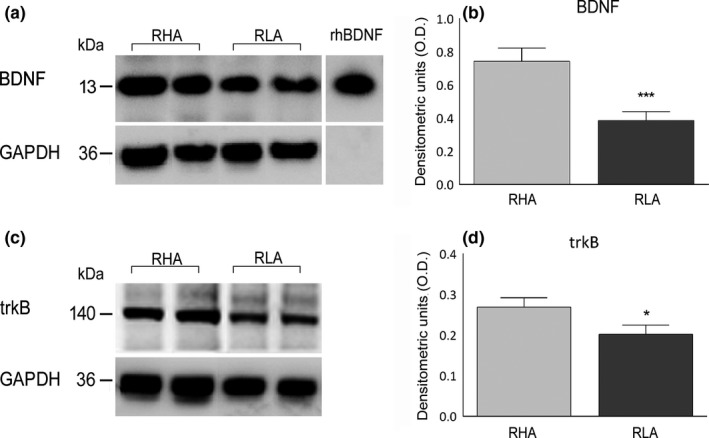
Western blot analysis of BDNF (a, b) and t rkB (c, d) in the dorsal hippocampus of RHA and RLA rats under baseline conditions. (a, c): shown are duplicate BDNF (a) and trkB (c) samples; (b, d): densitometric analysis of the BDNF/GAPDH (b) and trkB/GAPDH (d) band gray optical density (O.D.) ratios. Columns and bars denote the mean ± *SEM* of eight rats in each experimental group. *: *p* < .05; ***: *p* < .001 versus the RHA group (two‐tailed Student's *t* test for independent samples)

The anti‐BDNF antibody recognizes a protein band with a relative mw of about 13 kDa (Figure 2), in agreement with the reported mw of the monomeric form of the protein (Rosenthal et al., 1991). As shown in Figure 2, the molecular mass of the hippocampal BDNF protein is very close to that of the rhBDNF (Leibrock et al., 1989). In tissue homogenates from the dorsal hippocampus the relative levels of BDNF protein (RLA = 0.39 ± 0.05 vs. RHA = 0.74 ± 0.08) were 48% lower in RLA versus RHA line (*p* < .001; Table 1).

**Table 1:** Statistical analysis of BDNF‐ and trkB‐like immunoreactivity by western blot and immunohistochemistry in the hippocampus of RLA and RHA rat lines

Method	Hippocampal region		Marker	Line	Number of samples	*p* value	Mean	*SEM*	t, *df*
Western blot	Dorsal hippocampus		BDNF	RHA	15	.0008	0.7436	0..07909	3.761, 28
				RLA	15		0.3868	0.05241	
			trkB	RHA	15	.0446	0.2691	0.02286	2.102, 28
				RLA	15		0.2022	0.02218	
Immunohistochemistry	Dorsal hippocampus	Dentate gyrus	BDNF	RHA	12	ns	164.6	16.68	1.163, 22
				RLA	12		140.5	12.29	
			trkB	RHA	12	< .0001	259.3	11.28	5.951, 22
				RLA	12		169.1	10.12	
		CA3	BDNF	RHA	12	.0002	232.0	14.55	4.493, 22
				RLA	12		160.3	6.562	
			trkB	RHA	12	ns	115,5	4,679	0.672, 22
				RLA	12		120,9	6.591	
		CA2	BDNF	RHA	12	.0257	151.1	13.67	2.392, 22
				RLA	12		112.8	8.239	
			trkB	RHA	12	ns	128,6	3.990	1.114, 22
				RLA	12		137,0	6.416	
		CA1	BDNF	RHA	12	.0059	221.1	16.74	3.045, 22
				RLA	12		163.2	9.030	
			trkB	RHA	12	ns	132,8	2.513	0.194, 22
				RLA	12		133,6	3.473	
	Ventral hippocampus	Dentate gyrus	BDNF	RHA	12	.0382	187.6	20.21	1.746, 23
				RLA	12		136.0	11.75	
			trkB	RHA	12	ns	90.39	15.17	0.4256, 22
				RLA	12		82.52	10.57	
		CA3	BDNF	RHA	12	.0427	167.9	18.76	2.151, 22
				RLA	12		120.1	11.9	
			trkB	RHA	12	ns	34.79	4.267	0.3874, 22
				RLA	12		32.24	4.994	
		CA1	BDNF	RHA	12	ns	148.4	17.51	0.3259, 22
				RLA	12		155.6	13.86	
			trkB	RHA	12	ns	35.99	4.469	0.5312, 22
				RLA	12		40.20	6,543	

The anti‐trkB antibody recognized a protein band with a relative mw ≅ 140 kDa (Figure 2), consistent with the mw of the full‐length, biologically active form of the receptor protein (Klein, Parada, Coulier, & Barbacid, 1989). The relative levels of trkB receptor protein (RLA = 0.40 ± 0.05 vs. RHA = 0.68 ± 0.12) were 25% lower in the RLA versus the RHA line (*p* < .05; Table 1).

### 3.2 Immunohistochemistry

**Figure 3:**
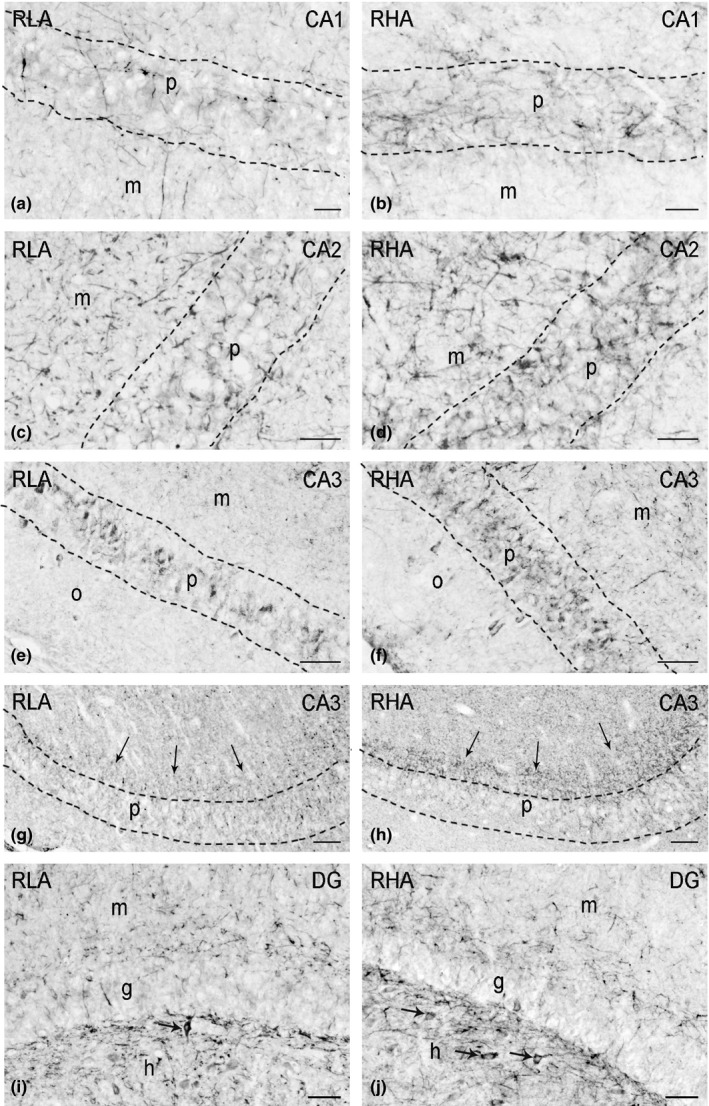
BDNF‐like immunoreactivity in the dorsal hippocampus of RLA (left column) and RHA rats (right column). (a, b): CA1 sector; (c, d): CA2 sector; (e–h): CA3 sector of the Ammon's horn; arrows in (g–h) point to labeled punctate elements in stratum lucidum; (i, j): dentate gyrus (DG); arrows point to BDNF‐labeled neurons. Dashed lines mark the boundaries of the Ammon's horn pyramidal layer. g, granule cell layer; h, hilus; m, molecular layer; p, pyramidal layer; o, stratum oriens. Scale bars: 50 μm

**Figure 4:**
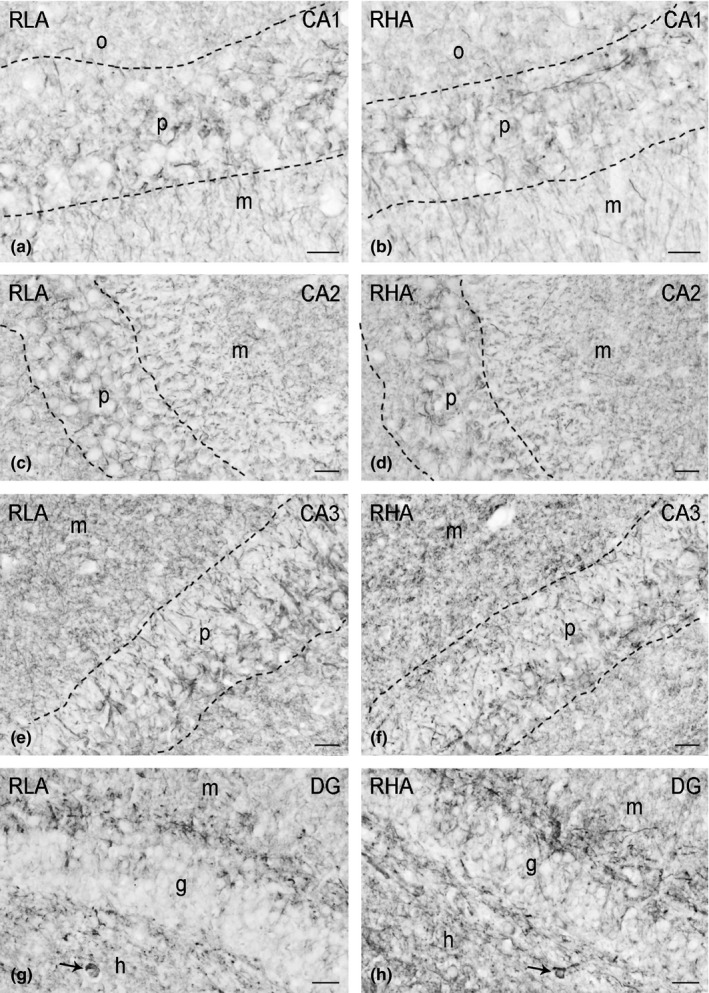
TrkB‐like immunoreactivity in the dorsal hippocampus of RLA (left column) and RHA rats (right column). (a, b): CA1 sector; (c, d): CA2 sector; (e, f): CA3 sector of the Ammon's horn; (g, h): dentate gyrus (DG). Dashed lines mark the boundaries of the Ammon's horn pyramidal layer. Arrows point to trkB‐labeled neurons. g, granule cell layer; h, hilus; m, molecular layer; p, pyramidal layer; o, stratum oriens. Scale bars: 50 μm

In tissue sections, labeling for BDNF (Figures 3, 6) and trkB (Figures 4, 6) showed an uneven distribution within the hippocampal formation. Both the BDNF‐ and trkB‐immunostainings mostly labeled neuronal proximal processes and nerve fibers distributed within the Ammon's horn and the dentate gyrus. A number of BDNF‐ and trkB‐positive cell bodies were also observed. Although BDNF‐ and trkB‐like immunoreactivities shared a similar distribution, they showed a differential density in the hippocampal subregions in both dorsal and ventral hippocampus.

**Figure 5:**
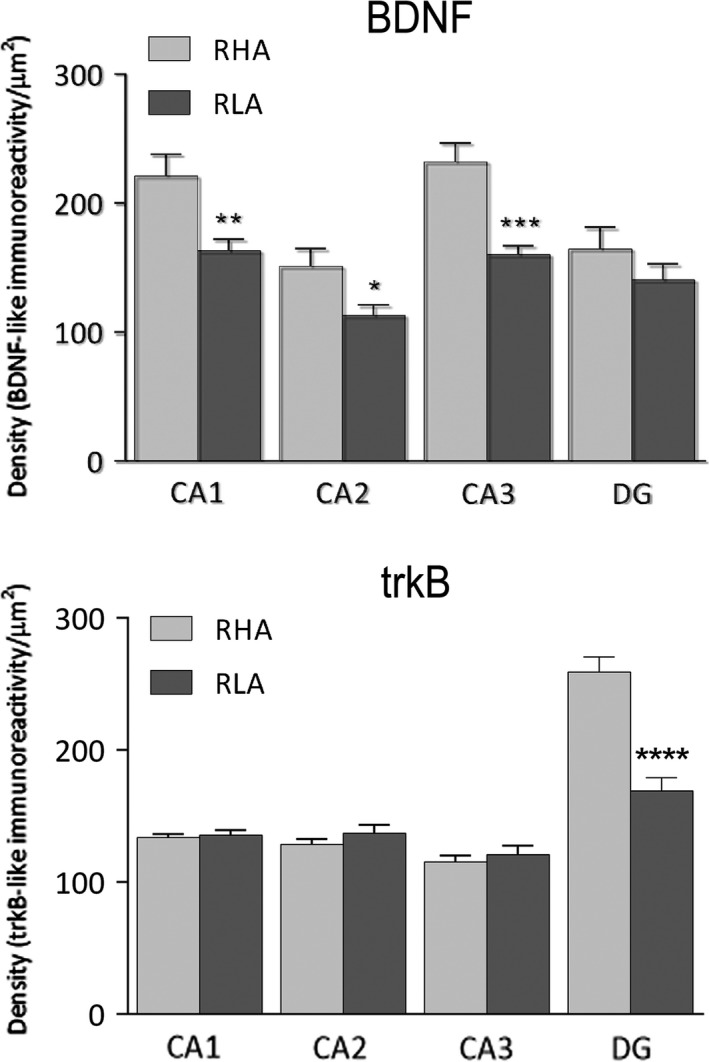
Densitometric analysis of BDNF‐ (upper panel) and trkB‐like immunoreactivity (lower panel) in the CA1‐CA3 sectors of the Ammon's horn and in the dentate gyrus of the dorsal hippocampus. Columns and bars denote the mean ± *SEM* of six rats in each experimental group in two different microscopic fields per each hippocampal subregion. *: *p* < .05; **: *p* < .01; ***: *p* < .001 versus the RHA group (two‐tailed Student's *t* test for independent samples)

**Figure 6:**
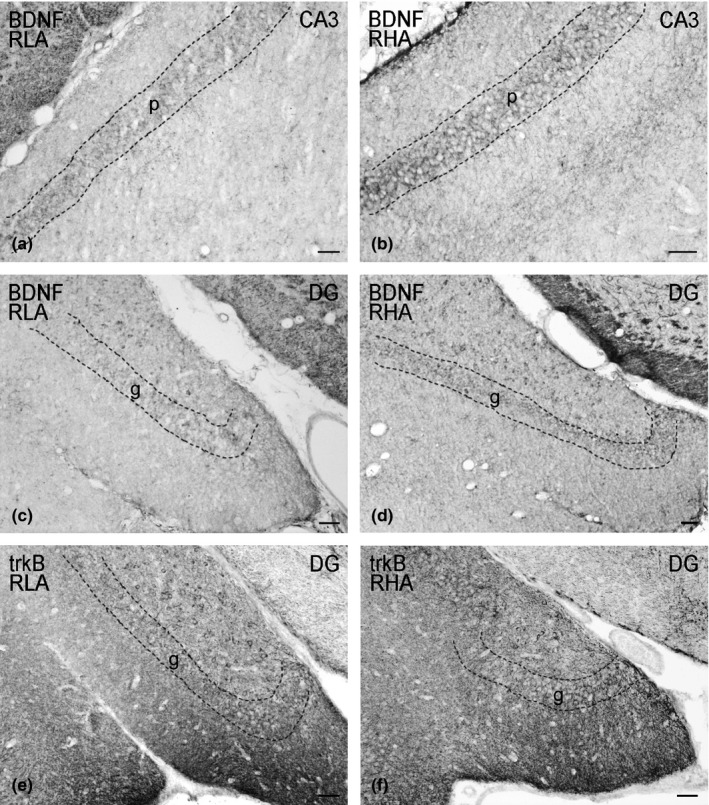
BDNF‐ (a–d) and trkB‐like immunoreactivity (e, f) in the ventral hippocampus of RLA (left column) and RHA rats (right column). (a, b): CA3 sector of the Ammon's horn; (c–f): dentate gyrus (DG). Dashed lines mark the boundaries of the Ammon's horn pyramidal layer and the granule cell layer of the DG. g, granule cell layer; p, pyramidal layer. Scale bars: 50 μm

**Figure 7:**
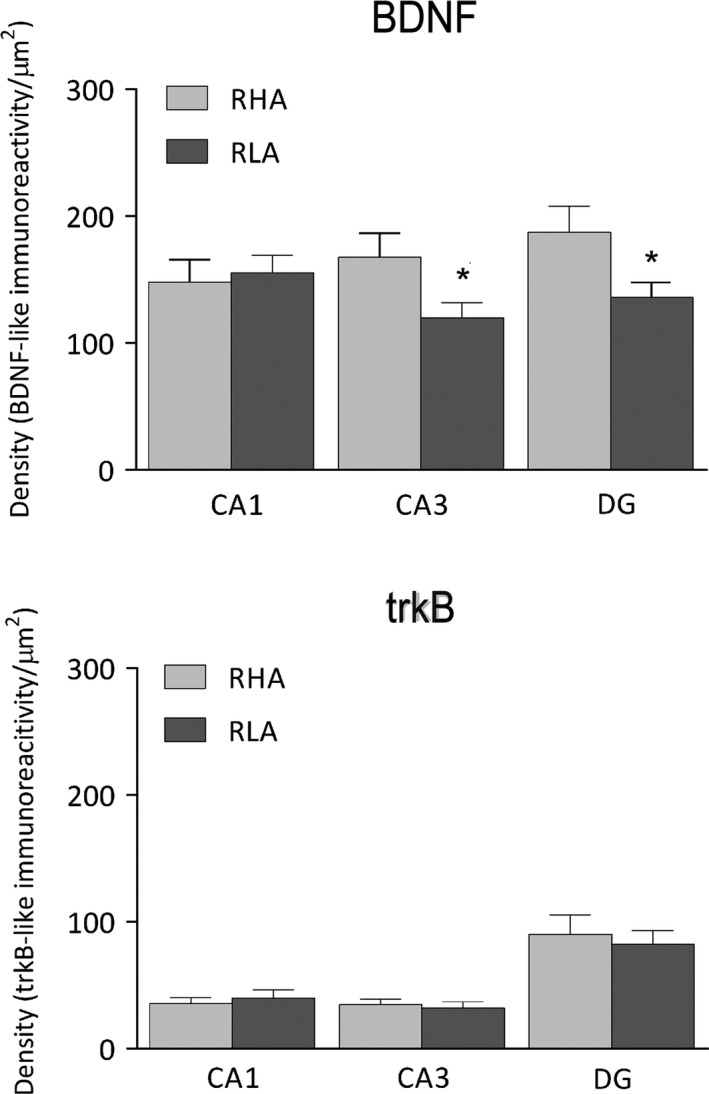
Densitometric analysis of BDNF‐ (upper panel) and trkB‐like immunoreactivity (lower panel) in the CA1‐CA3 sectors of the Ammon's horn and in the dentate gyrus of the ventral hippocampus. Columns and bars denote the mean ± *SEM* of 6 rats in each experimental group in two different microscopic fields per each hippocampal subregion. *: *p* < .05 versus the RHA group (2‐tailed Student's *t* test for independent samples)

BDNF‐immunostaining labeled extensive nerve fiber networks that were mostly distributed in the Ammon's horn, where labeled filamentous elements occurred in between the cell bodies of the pyramidal layer and in the molecular layer of the CA1 (Figure 3a, b), CA2 (Figures 3c, d) and CA3 (Figures 3e, f; 6a, b) sectors. Some BDNF‐positive perikarya were observed in the pyramidal, molecular and oriens layers (Figures 3e,f). In the CA3/CA2 subfield, a band of BDNF‐like immunoreactive punctate structures, that appeared to be denser in RHA than RLA rats, was observed in the stratum lucidum (Figures 3g,h). The dentate gyrus also harbored loose networks of positive nerve fibers and punctate elements (Figures 3i,j; 6c,d), distributed in the molecular layer, with increasing density from its outer third to the deep narrow band adjacent to the granule cell layer, and in the hilus. Some BDNF‐positive perikarya were observed within the granular layer, at the interface between the granule cell layer and the polymorphic layer, and in the hilus of the dentate gyrus (Figures 3i,j; 6c,d). Intensely BDNF‐like immunoreactive nerve fiber systems were also observed in the alveus and in the fimbria (not shown). Densitometric analysis (Figures 5; 7) further showed that BDNF‐like immunoreactivity (LI) was lower in the hippocampus proper of RLA versus RHA rats (see Table 1), with differences in the density of BDNF‐positive neuronal structures that, in the dorsal hippocampus, amounted to −34% in CA3 (RLA 160.3 ± 6.6 vs. RHA 232 ± 14.6; *p* < .001), −31% in CA1 (RLA 163.2 ± 9.03 vs. RHA 221.1 ± 16.7; *p* < .01) and −24% in CA2 (RLA 112.8 ± 8.2 vs. RHA 151.1 ± 13.7; *p* < .025), while in the ventral hippocampus were −28.5% in CA3 (RLA 120.1 ± 12.9 vs. RHA 167.9 ± 18.76; *p* < .05) and −27.5% in the DG (RLA 136 ± 11.7 vs. RHA 187.6 ± 20.21; *p* < .05).

TrkB‐immunostaining also labeled extensive nerve fiber systems, mostly appearing as filaments, short hollow tubules and coarse punctate elements. Positive elements occurred in both the Ammon's horn (Figure 4a–f) and the dentate gyrus (Figures 4g,h and 6e,f). In the latter, labeled structures consisted of filaments and punctate structures distributed in between the granule cell bodies, deep under the molecular layer, and, with lesser density, in the hilus (Figure 4g,h). Rare trkB‐immunolabeled neuronal cell bodies and/or their proximal processes were observed in the thickness of the Ammon's horn pyramidal layer (Figure 4e) and in the hilus of the dentate gyrus (Figures 4g,h; 6e,f). Densitometric analysis in the CA sectors of the hippocampus proper and in the dentate gyrus (Figures 5 and 7) revealed that line differences are limited to the dorsal hippocampus and particularly to the dentate gyrus where the density of trkB‐like immunoreactive structures was 23% lower in RLA than RHA rats (RLA 169.1 ± 10.12 vs. RHA 259.2 ± 11.3; *p* < .0001), while no significant differences were found in the Ammon's horn (see Table 1).

## 4 DISCUSSION

The main finding of the present study is that, under baseline conditions, the relative protein levels of BDNF and its selective receptor trkB, measured by WB, are significantly lower in the dorsal hippocampus of depression‐vulnerable RLA rats versus depression‐resistant RHA rats. Moreover, the densitometric analysis of immunostained brain slices revealed that the BDNF‐LI is lower in the Ammon's horn whereas trkB‐LI is lower in the dentate gyrus of RLA rats as compared to their RHA counterparts. These findings add experimental support to the neurotrophic hypothesis of depression, which postulates that decreased levels of BDNF in the hippocampus play a critical role in the pathophysiology of this mental disorder (Duman & Monteggia, 2006; Kozisek, Middlemas, & Bylund, 2008). Accordingly, by means of the same antibody used in this study, the relative levels of the BDNF mature protein in the hippocampus of the Flinders sensitive line of rats, a genetic model of depression, have been shown to be significantly lower than in their Flinders resistant controls (Elfving et al., 2010). Moreover, a decrease in the BDNF mature protein has been reported in the ventral hippocampus and frontal cortex of rats with a genetic deletion of the serotonin transporter (Calabrese et al., 2013). A selective deletion of the BDNF‐encoding gene within the mouse dentate gyrus also results in a reduction in antidepressant efficacy (Adachi, Barrot, Autry, Theobald, & Monteggia, 2008) and, conversely, the infusion of BDNF into the lateral cerebral ventricle (Hoshaw, Malberg, & Lucki, 2005) or the dorsal hippocampus (Shirayama et al., 2002) elicits antidepressant effects in behavioral models of depression. There is also growing evidence that antidepressant treatments may exert some of their therapeutic effects by increasing BDNF expression levels and affecting BDNF transcription in the hippocampus. Thus, (i) electroconvulsive brain stimulation, a treatment of choice for medication‐resistant depression, increases the hippocampal contents of BDNF and trkB mRNAs (Altar, Whitehead, Chen, Wörtwein, & Madsen, 2003; Angelucci, Aloe, Jiménez‐Vasquez, & Mathé, 2002; Nibuya et al., 1995), particularly in the dorsal hippocampus (Ploski et al., 2006), (ii) antidepressant treatment rapidly elevates the content of BDNF mature protein via posttranscriptional mechanisms, prevents the stress‐induced decrease in the hippocampal concentration of BDNF mRNA and counteracts the depression‐associated alterations in neural plasticity by normalizing BDNF in the rodent prefrontal cortex and hippocampus (Baj et al., 2012; Kozisek et al., 2008; Musazzi et al., 2009). In keeping with these experimental findings, postmortem samples from clinically depressed patients treated with antidepressant medications show increased BDNF immunoreactivity in the hilus, the dentate gyrus and the supragranular regions of the hippocampus as compared with antidepressant‐untreated controls (Chen, Dowlatshahi, MacQueen, Wang, & Young, 2001).

Interestingly, it has been shown that different antidepressant treatments, such as physical activity (Baj et al., 2012; Kozisek et al., 2008), the electroconvulsive treatment, and several antidepressant medications (Adachi et al., 2008; Alboni et al., 2010; Baj et al., 2012; Elfving et al., 2010; Kozisek et al., 2008; Marmigère, Givalois, Rage, Arancibia, & Tapia‐Arancibia, 2003) modulate differently the expression of BDNF in specific hippocampal subfields. Therefore, in the present study we evaluated the basal expression of BDNF and trkB in different subregions of the hippocampus of the Roman rat lines. The densitometric analysis of the brain sections revealed that in the dorsal hippocampus BDNF‐like immunoreactive structures show a lower density in the Ammon's horn, while trkB‐positive structures have a lower density in the dentate gyrus of RLA as compared with RHA rats. As for BDNF‐LI, such differences are also clear in the ventral hippocampus where both the CA3 sector and the DG show a lower density in RLA versus RHA rats, while the density of trkB‐LI does not vary among lines.

The lower levels of BDNF‐like immunoreactivity in the Ammon's horn of RLA versus RHA rats, especially in the CA3 sector, are consistent with the hypothesis that, in the RLA line, CA3 neurons may have a slower synthesis rate of BDNF. In the CA3 sector BDNF is known to be both locally produced and anterogradely transported along the mossy fiber axons of the granule cells in the dentate gyrus. Thus, the possible slower production of BDNF protein in RLA versus RHA rats, as also suggested by the lower density of BDNF‐LI in the dentate gyrus of the ventral hippocampus, may in turn lead to a reduced target‐derived support to promote the synaptic contacts with the mossy fibers. Interestingly, a selective increase in both BDNF mRNA and protein in the CA3 and CA2 sectors has been reported upon repeated antidepressant treatment (De Foubert et al., 2004). The significantly lower density of trkB immunoreactivity observed in the dentate gyrus of the dorsal hippocampus of RLA versus RHA rats further suggests that the functional tone of BDNF‐trkB signaling in the dentate gyrus may be less intense in RLA rats as compared to their RHA counterparts.

The immunolabeling of both BDNF and trkB in the dentate gyrus is localized to cell bodies and nerve fiber networks. The occurrence of BDNF‐containing granule cells is in line with the reported synthesis of BDNF mRNA in this hippocampal region (Phillips et al., 1991). However, the very small number of immunoreactive granule cells in our preparations does not allow us to evaluate possible quantitative differences in their occurrence between RLA and RHA rats. According to the neurotrophic hypothesis of depression, the biological substrate at the basis of the effectiveness of antidepressants is the integration of the newly born granule cells to the hippocampal neural circuitry (Baj et al., 2012; Duman & Monteggia, 2006) by establishing the typical synaptic connections of mature granule cells, i.e., to receive projections from the entorhinal cortex through their dendritic arbors extending in the molecular layer and to establish axo‐dendritic synapses with the CA3 pyramidal neurons (Amaral, Scharfman, & Lavenex, 2007). Interestingly, the dentate gyrus shows increased BDNF levels in the rat hippocampus after chronic antidepressant treatment (Nibuya et al., 1995; Shirayama et al., 2002), and studies in mice with selective BDNF‐encoding gene deletion in specific hippocampal subfields have demonstrated that BDNF expression in the dentate gyrus, but not in the CA1 sector, is essential for the effectiveness of antidepressants due to its possible involvement in supporting survival and differentiation of newborn granule cells (Adachi et al., 2008). Notably, BDNF/trkB‐LI labels other cell types in the dentate gyrus of the Roman rats, such as those located in the subgranular layer and in the hilus, some of which resembling the dentate pyramidal basket cells and the mossy cells described by Amaral et al. (2007), that may further contribute to the modulation of the hippocampal functional connectivity. In our preparations, at the level of the dorsal hippocampus, both BDNF‐ and trkB‐like immunoreactive nerve fiber plexuses appear to be denser in the inner third of the molecular layer, where the axons originating from the hilar mossy cells play a commissural/associational role in the rat dentate gyrus (Amaral et al., 2007 and references therein). The origin of the nerve fibers in the inner third of the molecular layer as well as the extent to which the possible commissural/associational projections contribute to the line‐dependent differences in trkB‐LI and, therefore, to the line‐related differences in BDNF signaling in the dentate gyrus, are not completely understood.

Studies on the BDNF targeting on hippocampal CA3 dendrites (Baj, Pinhero, Vaghi, & Tongiorgi, 2016; Baj et al., 2012; Righi, Tongiorgi, & Cattaneo, 2000) further suggest the possibility of concurrent mechanisms of BDNF‐mediated trophic support. Thus, upon the anterograde transport along the mossy fibers (Altar & DiStefano, 1998; Altar et al., 1997), the endogenous BDNF secreted along the neuronal activity may contribute to local mechanisms of trophic support that drive the accumulation of BDNF and trkB mRNAs in specific subcellular compartments of the CA3 pyramidal neurons (Righi et al., 2000). Further studies are warranted to assess the colocalization of BDNF and the trkB receptor protein and to characterize the hippocampal neural circuitry involved in the trophic activity of BDNF/trkB signaling in the Roman rats.

As mentioned above (see Introduction), RLA and RHA rats represent two divergent phenotypes respectively susceptible and resistant to develop depression‐like behavior under aversive environmental conditions. Thus, in the forced swim test (FST), RLA rats exhibit a depression‐like behavior characterized by greater immobility and fewer climbing counts when compared to their RHA counterparts, which exhibit proactive coping characterized by a shorter immobility time and more frequent climbing counts (Piras et al., 2010). Notably, subacute as well as chronic treatments with clinically effective antidepressant drugs decrease immobility and increase active behaviors (i.e., climbing and swimming) in RLA rats, but do not modify the performance of RHA rats (Piras et al., 2010, 2014). Therefore, the Roman rats may be used to identify and characterize neural substrates and mechanisms, such as BDNF/trkB signaling, underlying the vulnerability to stress‐induced depression as well as the molecular adaptations that mediate the resistance to such changes and the mechanisms of action of antidepressant drugs.

## DISCLOSURE

The authors declare that the research was conducted in the absence of any commercial or financial relationships that could be construed as a potential conflict of interest.

## AUTHORS' CONTRIBUTIONS

OG, MGC, MQ: Designed the study and wrote the manuscript; all authors contributed to experimental design; OG, MGC, MQ, and MPS: Interpreted the findings and revised the article; MGC, MAP, FS, and OG: Selected and bred the Roman rats; MPS, LP, MB, and MAP: Performed the sampling; MPS, LP, MB, and MQ: Performed the immunochemical experiments, image and statistical analyses; MQ: Performed the artwork. All authors read and approved the final manuscript.
